# Effects of Activity-Based Hospital Payments in Israel: A Qualitative Evaluation Focusing on the Perspectives of Hospital Managers and Physicians

**DOI:** 10.34172/ijhpm.2020.51

**Published:** 2020-04-18

**Authors:** Ruth Waitzberg, Wilm Quentin, Elad Daniels, Yael Paldi, Reinhard Busse, Dan Greenberg

**Affiliations:** ^1^The Smokler Center for Health Policy Research, Myers-JDC-Brookdale Institute, Jerusalem, Israel.; ^2^Department of Health Systems Management, School of Public Health, Faculty of Health Sciences, Ben-Gurion University of the Negev, Beer-Sheva, Israel.; ^3^Department of Health Care Management, Faculty of Economics & Management, Technical University Berlin, Berlin, Germany.; ^4^European Observatory on Health Systems and Policies, Brussels, Belgium.; ^5^Maccabi Healthcare Services, Tel Aviv-Yafo, Israel.

**Keywords:** Health Policy Evaluation, Hospital Payment Reform, Diagnosis-Related Group, Israel

## Abstract

**Background:** Since 2010, Israel has expanded the adoption of procedure-related group (PRG) based payments for hospitals. While there is a rich quantitative literature that assesses the effects of payment reforms on efficiency or quality of care, very few qualitative studies have focused on the impacts of diagnosis-related group (DRG)-like payments on hospitals from the perspective of hospital workers as change agents.

**Methods:** We used a qualitative, thematic analysis based on 33 semi-structured in-depth interviews with chief executive officers (CEOs), chief financial officers (CFOs), ward directors and physicians conducted in five public hospitals in Israel, sampled by maximum variation according to hospital characteristics.

**Results:** Interviewees reported that the payment reform led to organizational changes such as increased transparency and enhanced supervision. Interviewees also reported several actions in response to the economic incentives of PRGbased payment. These included (1) shifting activities to afterhours and using operating rooms (ORs) more efficiently to enable increased surgical volumes; (2) reducing costs by shortening lengths of stay and increasing cost-consciousness in procurement; and (3) increasing revenues by improving coding and selecting procedures. Moderating factors reduced the effects of the reform. For example, organizational factors such as the public nature of hospitals or the (un)availability of healthcare resources did not always allow hospitals to increase the number of cases treated. Also, conflicting incentives such as multiple payment mechanisms or underpricing of procedures blurred the incentives of the reform. Finally, managers and physicians have many other considerations that outweigh the economic ones.

**Conclusion:** PRG payments affected the organizational dynamics of hospitals and changed decision-making about admission and treatment policies. However, such effects were moderated by many other factors that should be considered when shaping and analyzing hospital payment reforms.

## Introduction

Payment methods (PMs) to healthcare providers create incentives that influence their behavior.^1^ Reforms of PM can lead to changes in provider decision-making regarding the policy of admission or treatment of patients.^2^ Many high-income countries, including Israel, adopted diagnosis-related groups (DRGs) and their local variants as the basis for hospital payment during the late 1990’s aiming to increase efficiency and transparency.^3^

Between 2010 and 2014, Israel expanded the use of procedure-related group (PRG) based payments to hospitals, its local variant of DRGs, replacing part of the traditional per-diem (PD) payments for about 150 elective procedures in the clinical fields of General Surgery, Gynecology, Ophthalmology, Orthopedics, Otorhinolaryngology, and Urology.^4^ PRGs differ from DRGs in that they categorize patients primarily based on type of treatment (surgical procedure) and not on diagnosis, and they are not adjusted for case-mix.^5^ The main objectives of this PM change were to reduce cost-price gaps thus improving fairness of hospitals payment, and to strengthen the capacity of the Ministry of Health (MoH) to supervise and control hospital activities.^4^

Israel has a National Health Insurance system with 4 competing not-for-profit health plans (HPs) responsible for the provision of healthcare to their members. They provide primary care, may contract with specialists for outpatient care, and purchase services from hospitals. The general non-profit hospital sector is composed of hospitals owned by HPs, non-governmental organisations (NGOs), the MoH or municipalities, and represents 93% of acute-care beds in Israel. Non-profit hospitals are often referred to as ‘public hospitals,’ as they are subject to uniform MoH regulations, certificate of needs, maximum price-lists and other constraints. They can only treat publicly-funded patients (except in Jerusalem, due to historical reasons), while private hospitals can treat privately-funded patients too, and therefore select their patients.^5^ The Israeli hospitals are some of the most crowded among the Organisation for Economic Co-operation and Development (OECD) countries, functioning with approximately half the rates of acute care beds and nurses per population, one of the shortest average length of stay (ALoS) and substantially higher bed occupancy rates compared with OECD averages.^6,7^

Sales of services represent 88% of hospitals’ income, of which between 33% and 50% come from PRG-based payments, about 40% from PD and the remaining (10%-25%) is fee for service (FFS).^8^ The method of payment and tariffs are set by the MoH, and cover hospitals’ marginal costs and some fixed costs such as nurses and physicians’ salaries during regular working hours (7 am-4 pm). MoH-owned hospitals also receive prospective subsidies from the government to cover part of other fixed costs such as infrastructure and equipment. Hospitals negotiate with the MoH retrospective subsidies if they are in deficits, and these typically represent the other 12% of hospitals’ income. There are two major income constraints to non-profit hospitals. The first is an annual cap on revenues from each HP to each hospital. If a hospital provides more services to the insured of a particular HP than the cap threshold, the HP pays only a percentage of the full cost, for those services exceeding the cap. The second is negotiations between HPs and hospitals that can set alternative reimbursement contracts, which supplant the official cap, and entail discounts that vary across HPs and hospitals.^5,9^ Usually small hospitals or those located in areas with high density of hospitals give more discounts to HPs in order to attract them to refer patients.^8^

Elective procedures performed in after-hours are paid PRG, and each hospital can decide whether to work in after-hours and what procedures to perform. Payments are made to the hospital. Wards do not have specific budgets or income, and depend on consent from the MoH and the hospital management to be able to purchase equipment, open new beds or hire more staff.

Managers, physicians, and nurses are salaried employees. Salaries are set nationally and are subject to salary agreements between professional associations (eg, Israel Medical Association) and the Ministries of Health and Finance. Salary levels are primarily a function of role and seniority, years of work experience and the number of shifts worked; therefore, income does not depend on the type or amount of procedures performed. Physicians are sometimes allowed to work extra hours after their regular shifts and hospitals may decide to provide additional FFS payments to physicians for work performed during after-hours.^5^

Many studies have evaluated the effects of the introduction of DRGs on hospital efficiency, eg, measured by length of stay (LoS) or volumes, and quality, eg, measured by mortality or readmissions.^10^ While in many countries LoS decreased, no consistent impacts were found regarding volumes and quality of care.^10-14^ In Israel, studies found that the effects of the adoption of PRGs on hospital activity were rather weak. Shmueli and colleagues^15^ explored the short-term effects of the early adoption of PRGs for 5 procedures in 4 hospitals in 1990 and found decreases in LoS but no clear trend in changes of volumes and quality of care. Waitzberg et al^9^ examined the short and long-term effects of the later expansion of PRG payments in 2010-2014, and found no significant impact on ALoS or number of discharges when analyzed at the ward level.

While there is a rich quantitative literature that has assessed the effects of DRG reforms on indicators of efficiency, very few qualitative studies have focused on its effects in hospitals as organizations, and from hospital workers’ perspectives as change agents.^16,17^ A few studies explored hospital senior managers’ and middle managers’ perspectives about the adoption of DRGs in England and Canada.^18-20^ Other studies examined physicians’ attitudes related to DRGs regarding ethical issues, conflicts, and commercialization of decision-making.^21-23^ However, to our knowledge, no study compared perspectives of different hospital workers, from senior executives to practicing clinicians. Information from a range of hospital workers as informants may shed light on changes of hospital culture, behavior and treatment policy that are not captured by quantitative indicators. For example, improvement of unmeasured quality of care, but also unintended consequences, such as selection of profitable patients and/or procedures, and over-treatment or early discharges.

In this study, we examined the perspectives of hospital workers of various levels about the expansion of PRG payments in Israel that occurred between 2010 and 2014. Particularly, we explored what economic incentives this PM reform created, how it affected admission and treatment decision-making, clinical practice, what changes occurred and how hospitals as organizations responded to the payment reform. We also analyzed how managers transmit the new economic incentives and considerations to physicians and how they embrace (or not) these new “rules of the game.” These issues remain underexplored in countries that reformed or intend to reform their hospital PMs.

## Methods

### Study Design and Participants

This qualitative study is based on *thematic analysis*, as we intended to generalize the experiences reported by employees of the sampled hospitals and to contribute to a deep understanding of other health provider payment reforms, both in Israel and internationally. Thematic analysis is suitable to applied research, as it identifies patterns of meaning across qualitative data in order to answer a defined research question.^24,25^

We selected study participants from 5 public hospitals sampled to provide maximum variation according to type of ownership (public/NGO/HP), location (center of the country, ie, close to Tel Aviv/periphery), and size of hospital (big/small). We chose these characteristics to capture diverse perspectives on how economic incentives affected hospitals, and how organizational culture and (dis)economies of scale played a role in this process. We excluded (private) for-profit hospitals because they are not subject to the MoH’s price-list and therefore were not directly affected by the PRG reform.

In order to supply rich and varied information on attitudes and perceptions regarding the adoption of PRGs by hospitals as organizations and their agents (ie, their staff), we selected respondents of different roles in each hospital. These included chief executive officers (CEOs), chief financial officers (CFOs), ward directors and physicians who worked in inpatient surgical wards for which many PRG codes were created between 2010 and 2014, such as ophthalmology, orthopedics, urology, and general surgery.^4^ We chose not to interview nurses and other hospital staff as they were less involved in decision-making related to admission and treatment policies.

### Data Collection

Data were collected through standardized open-ended in-depth interviews. We initially built the interview protocol based on our research questions. The protocol included the following main topics: How did the change in payment mechanism affect the interviewee’s way of working, his role, and his relationship with other hospital workers? Did he transmit or receive new messages? What messages? And how did he transmit or receive these messages? We further modified our protocol according to the interviewees’ reactions and the way they understood the questions, eg, we added questions about measurement of activities and “success,” and about priority-setting. Please see electronic Supplementary file 1 for the last version of the interview protocol.

All interviews were conducted in Hebrew and took place at the participants’ offices or other place of their preference. Interviews lasted between 30 to 90 minutes, all were recorded and transcribed; data collection and analysis took place concurrently. The authors did not take field notes. All interviewees signed an informed consent form before the interview and participants were assured full confidentiality. Most interviewees had no previous relationship with the researchers, and all were aware that the study was part of the main author’s PhD.

### Data Analysis

The analysis included triangulation: RW and ED read and coded all interviews in parallel, continuously opening and developing codes independently and always comparing and improving the codes together. Periodically RW, ED, YP and DG cross validated and reconciled the coding, and created and modified categories, as they changed during the analysis process. Finally, RW, DG, and WQ consolidated categories into three major themes. The analysis was done with the Narralizer^®^ software. It involved data familiarization, coding, building themes, and revising them. The analysis approach was both deductive, as codes and categories initially built from the research questions, based on economic and organizational theory; and inductive, as they were further refined and recreated emerging from participants’ narratives and discourse. The original citations in Hebrew were translated to English and their accuracy were validated by two of the researchers (RW and DG). Data saturation was discussed among RW, ED, DG, which led to the decision of when to stop conducting new interviews.

## Results

RW, ED, and DG interviewed face-to-face 33 hospital employees from December 2017 to August 2018. We invited 53 hospital employees to participate in our study via email and by phone, of whom 20 refused or did not respond. Our study participants included 4 hospital managers, 6 CFOs, 11 ward directors, and 12 physicians. It is important to note that in Israel hospital CEOs are physicians themselves, but do not operate as clinicians, while ward directors act both as managers and practice medicine. All varied in ethnicity, age and seniority in practice. Only 1 interviewee was female. Participants’ main characteristics are presented in Table.

**Table:** Participant’s Main Characteristics

**Hospital Characteristics**	**No. of Participants**
Hospital location	
Periphery	18
Center	15
Hospital size	
Big (> 500 beds)	29
Small (<500 beds)	4
Hospital ownership	
Public	8
HP	14
NGO	11
Interviewee characteristics	
Age, mean (range)	53 (39-67)
Years in practice, mean (range)	10 (0.5-27)
Role	
CEO	4
CFO	6
Ward director	11
Physician	12
Ward	
Orthopedics	12
General surgery	5
Cardiovascular surgery	2
Ophthalmology	2
Urology	2

Abbreviations: CEO, chief executive officer; CFO, chief financial officer; HP, health plan; NGO, non-governmental organisation.

The findings from this qualitative analysis are based in part on the interviewees’ direct responses to the questions they were asked and in part on subjects they raised by themselves. Some codes were determined in advance, based on the research question, while others emerged from the interviews. Respondents of the same role or type of ward gave more consistent responses to the interview questions than respondents from the same hospital holding different positions. For example, ward directors of urology in different hospitals were more aligned in their responses than ward directors of general surgery, CFO and CEO of the same hospital. It seems that the PRG reform affected the different workers and wards in different ways.

We found two themes that reflect the effects of the payment reform on hospitals as organizations – organizational changes and responses to PRG incentives – and one theme that summarizes moderating factors that counterbalanced these effects. The themes, subthemes, and categories are shown in Figure. A description and explanation of each follows.

**Figure:**
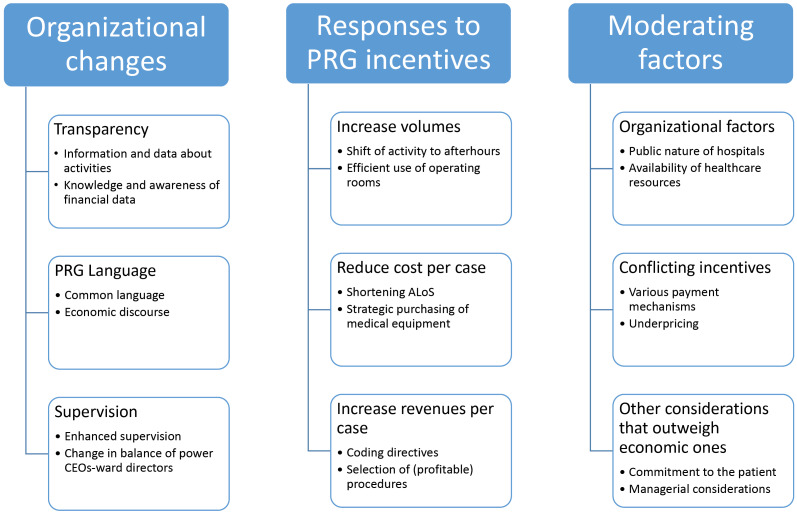
Themes, Sub-themes and Categories That Emerged From the Analysis. Abbreviations: PRG, procedure-related group; CEOs, chief executive officers; ALoS, average length of stay.

### 1. Organizational Changes

Study participants reported three main organizational changes in hospitals as a result of the introduction of the PRG payment system. The first was an increase of transparency and improved information availability, which influenced the organizational culture. The second was a creation of a new common language with payment codes that enabled an economic discourse. The third was an enhanced supervision by various players in the hospital market, and changes in the balance of power between CEOs and ward directors.

#### Transparency

With PRGs, hospital revenues depend on precise coding of procedures, therefore, the quality of medical documentation has improved and consequently transparency and data about hospital activity and finances has increased.

*“I do not know if it affected the behavior, it just made a lot of order, because all these codes… in the past we did not have codes. All [the information] was in ‘days,’ you could not know what was being done here at all”*(CFO 2221).

Detailed data about activities and a precise payment tariff known prospectively also enabled hospital managers to calculate their financial balance, increase efficiency and plan their wards’ activities ahead.

*“When I look as a manager, of course I want most of my surgeries to be PRGs. It is also a preference from the hospital management perspective, because it is easier to calculate its costs, easier to check our efficiency”*(Ward director 2845).

With more data about activities, the knowledge of financial data also increased among all players, particularly ward directors. Transparency was a means to change ward directors’ considerations, as they became more aware about their wards’ financial activities.

“*We know, for example, that if we operate on a 67-year-old woman with a fractured hip within 48 hours we get 60, 60 something thousand shekels. Not us, the hospital. If it’s not done on time, it’s only 45000 shekels. So, we know that, in general, we have to make an effort to get it done within 48 hours”*(Ward director 2431).

#### Procedure-Related Group Language

PRGs created a common language for hospital managers and physicians because they condensed the confusingly large number of different (individual) patients treated into a manageable number of medically meaningful and economically homogenous groups. Each PRG is characterized by a particular payment code and groups together patients with similar medical or surgical procedures and with relatively similar costs. All procedures included in a PRG are paid based on a fixed maximum price, which enables hospital managers, ward directors and physicians to analyze the costs and the profitability of different clinical activities. The new common language, ie, the PRG codes, facilitated a new type of discourse between managers and physicians. Physicians and managers started using the same terms (PRG codes) and the clinical discourse became more precise:

*“The PRG’s help is simple, you know, it makes sense. You can categorize. Now, there is a code, and we have a common language. Because you know that every procedure can have endless names. Some time ago there was an attempt to create PRG codes for laboratory tests. And one of the first things they discovered, was that the same bacteria had eight names. And everyone wrote it differently, and it was impossible. It was crazy. At first, they had to decide how to call each bacteria, and attach to each bacteria one single name and one single code. Now the PRG really makes order.”*(CEO 1111).

PRG codes made it possible for managers (CEOs, CFOs, and ward directors) to adopt an economic discourse and analyze (un)profitability of procedures or wards’ activities, which consequently increased the awareness of economic implications of clinical activities:

*“I can tell you that the management has clear messages, and they ask me: ‘Increase volumes, increase output, do more this and that.’ They do supervise as I supervise my ward so I can know what’s going on”* (Ward director 2431).

Nevertheless, transparency and the economic discourse remained mainly among managers, as they usually choose not to pass on information about economic aspects of the activity to practising clinicians. They believe that clinicians’ decision-making and clinical considerations should not be influenced by economic incentives, and they as managers are the ones with the dual responsibility, clinical and financial.

*“We didn’t pass it [these messages] on to the [physician] team. And I think that’s not right either. I mean it is wrong that doctors and nurses know, and because of that they will behave differently, because of the payment [PRG]. It is immoral”*(CEO 4111).

This poor communication and lack of transparency from managers to physicians ended up having the opposite effect: messages and changes got through without explanations or rationale, and made physicians suspicious and uncooperative with these changes.

*“Once we did after-hours surgery. I don’t know why they stopped it. I can’t explain the hospital’s considerations as to why not to allow after-hours surgery. It seems odd to me, but there isn’t any. I’m telling you my guesses and I don’t know where the truth lies. I haven’t a clue. I don’t understand the logic behind it, that the hospital allows entire ORs to be empty for two-thirds of the day”* (Physician 1341).

#### Supervision

Numerous study participants described increased supervision and improved transparency as other important effects of the introduction of PRGs. It seems that the PRG payment reform succeeded in one of its main objectives, ie, to increase supervision capacity and control by enabling greater transparency of activity and expenditure. For example, the CFO of a hospital has begun to supervise the departments’ activity more closely, while he himself is under closer supervision by the HPs. The MoH started supervising hospitals more closely, including its own hospitals.

*“We watched orthopedics non-stop in order to make sure they were not sending [urgent] people home and bringing them back electively, because we lose money that way. […] We are supervised by the health plans. They supervise charges and they are not suckers”* (CFO 1221).

Knowledge about costs and profitability of PRGs has empowered ward directors and senior physicians, and let them re-organize the work-plan of their wards. It also enabled a new form of negotiation for resources with hospital CEOs, and increased ward directors’ autonomy despite the increased supervision:

*“They [management] used to tell me, ‘Oh, you lose [money] on cataract surgery,’ but I said “Excuse me, how do you know? You are telling me I lose, but I calculated it. Because now I have PRGs. So now I know what I’m getting and I know what the expenses are: how much does a cataract set cost me, all the disposables I know. And the surgeon and nurse [cost] calculation. They [management] would prevent me for doing cataract surgery because I was losing money, which is nonsense. The CEO came and I told him to check. After he checked, he told me to operate as much as possible”*(Physician 3741).

### 2. Responses to PRG Incentives

The second theme that emerged from interviews were responses to the new economic incentives of PRGs. These responses attempt to increase hospitals’ profitability through various changes in patients’ admission and treatment policies, and are expected effects of DRG-based payment according to the existing literature: increasing volume of care (when procedures are not underpriced), reducing costs per case, and increasing revenues per case.^11^

#### Increase Volumes of Care

Hospitals were incentivized to carry out more (profitable) procedures, and this occurred mainly in orthopedics and urology wards, where, according to our interviewees, procedures were usually priced approximately equal or above marginal costs compared to general surgery or cardiovascular wards, where procedures were underpriced. Two main mechanisms enable public hospitals in Israel to increase activity. The first is improving the efficient use of operating rooms (ORs), mainly through pressure, which was not always welcomed by physicians:

*“They [management] measure OR times, what percentage of surgeries started at 08:00? Within OR working hours from 08:00 am to 3:00 pm, how long had an OR worked? How long were the breaks? The management wants things that are conceptually impossible. They declare from their ‘tribune’ that they want 0% cancellations, and 100% utilization”*(Physician 1343).

The second mechanism to increase volumes is shifting procedures from regular working hours (usually from 7:00 am to 4:00 pm) to after-hours:

*“[Volume of] this surgery has grown very significantly here because it has a PRG price. And what benefit does it gives us? We can do it in afterhours as well. Because we have the ability to pay [physicians] out of the price, and perform more of it. The element of afterhours is significant”* (CFO 2221).

The positive spillover effect of the increase in volumes of activity was the shortening of waiting times for some elective procedures. Interviewees did not mention changes in referral thresholds. This is mainly because patients are referred to hospitals by outpatient physicians:

*“If you came to a ward, you would see that almost 50% [of the patients] were waiting two days, three days, sometimes a week for an operation. Today you do not see it. Today this department is like a train station. People come and go. So now, we hardly have to deal with queues thanks to [the fact] that throughout the system, the hospital has given us this platform of after-hours surgery to solve the problem of hip fractures that can’t wait”*(Ward director 1431).

Compared to managers, physicians’ perspectives regarding increases in volumes of care were less optimistic. Some physicians also raised a downside of increasing surgical activity too much, at the expense of other activities such as research. Their discourse was careful and full of reservations, and it required courage for them to criticize their managers.

*“The ward is managed in such a way that there are measures, you want the ward to bring a certain amount [of patients], because the more you operate, the more money you bring to the hospital. The more money you bring, the more highly regarded you are, and then you know, there’s the trickle-down effect and the department head expects every unit to bring in a certain amount of work. The price is that, of course, in areas that are less profitable, then, you know, as I understand it – I could be wrong – but can be shoved aside, I do not know, I may be wrong in what I say… It [increasing surgical activity] has a price. There is a loss of knowledge, that is to say, not everything can be quantified, and the director quantifies things in numbers, quantities, and other things that fall a bit. I mean how much research does the department do now?”*(Physician 2442).

#### Reduce Costs Per Case

Another economic incentive created by PRG payments was to minimize costs per procedure, eg, by shortening LoS or being more careful with purchasing of materials and equipment needed for surgeries. Shortening LoS was possible to a limited extent, and was seen particularly in orthopedics wards, one of the most profitable wards across all hospitals. It was widely stressed that physicians and managers shortened LoS and responded to other economic incentives only up to the point where it did not threaten the quality of care or patients’ health, and never beyond that:

*“If a certain number of hospital days is defined [by the hospital] for you for a PRG, then you make sure not to exceed them – that’s to say, as much as possible. If there’s a medical need, then of course, you do, but the emphasis is really on meeting these criteria”*(Physician 2442).

Some respondents reported that shortening LoS occurred, but not necessarily because of the PRG payment. Other factors such as technological advancement or more OR hours contributed to the shortening of LoS, although they might be related to the fact that PRG paid procedures are prioritized in hospitals.

*“If you ask me directly why the LoS decreased, I think it has many reasons. One, the technique; two, greater availability of OR; three, because the more you operate, the better skilled the staff becomes, and there will be fewer complications, fewer readmissions, fewer long admissions”* (Ward director 2431).

Another means to reduce costs per case is purchasing lower cost material that has the same quality and avoiding waste when using disposable material or equipment.

*“If I have PRGs, I know my revenues and my expenditures. And I want to show that I am not a spender, that we don’t waste. So, I say: ‘don’t open the threads before I tell you,’ any thread costs $10, ‘because maybe I will prefer another thread?’ The nurses ‘whoosh,’ they often open up to me, they think they know”* (Physician 3741).

#### Increase Revenues Per Case

Another reaction to the new economic incentives was to increase revenues per case. The easiest way is to improve the coding of activities. Managers emphasize the importance of precise coding of activity, not only in order to improve data and supervision, but as a means to receive the most adequate payment. However, when requests to change behavior, such as the coding directives, reached physicians without the proper information about the reason for the change,they were less cooperative to embrace the new requests.

*“We get requests, guidelines, and directives from the hospital management to code more accurately, as if before [PRGs] we didn’t even notice. Now they have emphasized it and we do the coding. There are people who check after us what we coded and whether we did it right. Because, you understand, when you are in a hurry, when you do not have time to look through all 10 procedures that you have done, you give one heading. That’s it! Most important is that you have written everything. How it is coded? What do I care?”*(Physician 1343).

The second mechanism to increase revenues is selection of elective procedures, giving preference to PRG-paid (profitable) procedures both in the morning shift and in afterhours, or changing the method of allocating patients from the emergency department to inpatient wards. Again, this occurred mainly in orthopedics and urology wards, as procedures were overpriced.

“*If I perform an operation with PRG, then the economic environment of the hospital, management, administration, like me a lot, they can give me more ORs, give me ORs in the afternoon. If the procedure is not PRG-paid, they don’t like me so much”*(ward director 2845).

Hospitals have incentives to prefer profitable PRG-paid activities, ie, overpriced procedures and low severity patients, for after-hours activities because they have to contract additional staff for after-hours surgery, which is reimbursed on the basis of FFS. Severe patients or underpriced procedures are performed in the regular working hours, as salaries and costs during regular working hours are paid independent of the activities performed. Moreover, in after-hours activities, only the personnel directly involved in the procedure is hired and paid. Other emergency care is less available than in the morning shift. If a complication occurs, there is more risk in the after-hours than in the morning shift.

*“[We’re] very strict about selecting procedures for after-hours surgery – [we choose] anything that can be done routinely and with minimal risk of complications, such as [the case of] a young woman with gallstones, with no complications, generally healthy, with no history of surgery, whatever can be done in 15 minutes with minimum risk to the operation. [She’s] a good candidate for after-hours surgery”*(Physician 1342).

Despite these responses, some ward directors and physicians were somewhat reluctant to talk openly about incentives for selection, and did not always agree with the hospital’s objective to increase income.

*“Prostatectomy is a PRG-paid surgery, it is profitable and I can see a ward director prioritizing it: ‘We want to do as much as we can.’ And then, patients who are sometimes even more urgent can be pushed aside because of this”*(Ward director 4831).

### 3. Moderating Factors

Despite the various effects that occurred with the adoption of PRGs, respondents also reported important factors that moderated or even curbed the impacts of the reform and counterbalanced PRG’s new economic incentives. These moderating factors shed light on the complexity of hospital behavior, which cannot always react to payment reforms or respond to incentives. Moderating factors also unpack managers’ and physicians’ broad environment and considerations when making decisions regarding patient admission and treatment. We found organizational factors, concomitant conflicting incentives, and other important considerations that dampen the economic incentives.

#### Organizational Factors

The public nature of the hospital market represented an important factor of hospitals as organizations that moderated change. Public hospitals cannot directly select patients, as it is difficult to refuse to perform a financially unprofitable activity or admit complex high-risk patients.

*“Because private hospitals have the privilege of choice. I do not have this privilege: who comes to the emergency room is treated. I always tell my doctors: this is not a Rotary Club, where you can say ‘this one [patient] I get, this other one I do not get’”*(Ward director 4431).

The intensity with which hospitals reacted to the reform depended on the (un)availability of healthcare resources, such as medical, nursing and other staff, ORs and beds, medical equipment, imaging technology, and post-acute care capacity. Ward directors and physicians explained that their response to the incentives of PRGs did not depend solely on themselves. For example, in order to increase the activity in a given ward, it is necessary to coordinate with the OR staff, nurses, cleaners, and anesthesiologists, and there has to be a vacant bed in the department. Early discharge of a patient may depend on coordination with the rehabilitation unit, and on continuity of care in the community.

*“We are dependent on the next stop after us [to reduce lengths of stay]. The matter of transferring [the patient] to rehabilitation, to some institution, that’s not something we have much influence over”*(Ward director 2431).

#### Conflicting Incentives

The hospital accounting system is complex, includes diverse methods of payment apart from PRGs and multiple financial incentives that sometimes collide. For example, hospitals receive PD payments, retrospective subsidies, capping on yearly income, discounts from HPs and agreements that replace PD or PRG payments. These different types of payment exist simultaneously, thus creating conflicting incentives that might blur the effects of the PRG reform, and end up moderating hospitals responses.

*“We are told that there are all sorts of things, caps, discounts, and the money goes to the ‘Kremlin,’ that is the health plan management. And the ‘Kremlin’ splits the money and the hospital doesn’t really see what it earns”*(Physician 1441).

Some features of the accounting system such as retrospective subsidies or the agreements with HPs, might even reduce the financial responsibility of hospital managers. This moderating factor was greater in small hospitals, which had tighter agreements with HPs (ie, alternative reimbursement contracts). These agreements determined the total amount of income a HP would pay to the hospital per year, and sometimes even the amount of procedures a hospital was obliged to provide to the HP. Managers explained that they did not really see PRG payments, but a kind of global budget, and therefore they were less responsive to economic incentives of PRGs than big hospitals.

*“In the agreements [with health plans], there is a number of particular procedures we must perform. For example, small traumas for which the payment are pennies, but if it is required in the agreement, then we do them”*(Ward director 4331).

A second type of conflicting incentives of PRGs is inappropriate pricing of procedures. PRG tariffs sometimes do not accurately reflect costs, and in many cases are underpriced. In such a case, there is no incentives to increase activity:

*“In a procedure it is necessary to put in a very expensive biological net. We stopped doing the procedure, because they [MoH] did not price the net in the PRG tariff. The hospital says ‘I am sorry I can’t spend 30000 shekels on a biological net.’ In the end, they [MoH] take the wind out of the sails. In the end, you say, all of this PRG reform wasn’t worth it because I lost money”*(CFO 2221).

#### Other Considerations That Outweigh the Economic Considerations

In addition, there are many other considerations that collide with the economic ones, and therefore moderate the responses of hospitals to the economic incentives of PRGs. These considerations include, for example, clinical considerations, or the commitment to the patients and preferences, ethical considerations, prestige, or the need to train young physicians.

*“I explained to him [the CEO] how much it [the procedure] costs, and it costs a lot of money. And he told me ‘the money should not interest you, I want to see the results, and if it does good for patients, then the hospital will do it. This is a very special and advanced procedure, which is not performed anywhere else.’ And we did it.* […]* As a ward director, I determine the variety of surgeries that my ward wants to do. The first criterion is to provide a public service. The second, is education of young doctors, interns and students who need to see a certain variety of procedures. Within all procedures we have, if there are procedures that are not interesting, I will put them in low priority and maybe not even do them. This hospital does not have to do all the procedures. This is a giant hospital, it is a huge semitrailer, we have to put a lot of weight on it, containers. We should not put a box on it and send it for a ride, that is how I look at it. There are enough smaller hospitals in our area that can do these other smaller procedures, and can do them well. We need to focus on the big things”*(Ward director 2845).

In small hospitals, usually located in remote areas, the personal commitment to patients and their families was particularly important, as hospital employees were well acquainted with them.

*“Sometimes the family asks: ‘We do not know how to get along at home, leave him another day [in the hospital],’ we take them into consideration. Even when the ward is overloaded. If possible, we take it into consideration. This is the only pressure that affects the team that is not really a medical consideration”*(CEO, 4111).

Different hospitals had similar orders of priorities regarding what and when to treat their patients. Usually, priorities were set according to needs, type of illness, clinical urgency and other medical considerations that outweigh economic considerations. Overall, responses to economic incentives existed in limited situations where elective procedures could be delayed, changed or moved to other shifts.

*“Our top priority is cancer patients; next come patients whose quality of life we can affect. Let’s say in the third place, people with a hernia, and that’s when the operation is not life-saving but affects the quality of life to some extent. And, in between there are trauma operations, obligatory operations, complications in the internal (general) medicine wards, someone who’s had an accident and needs surgery, so that’s more or less the priorities*” (Ward director, 1331).

## Discussion

In this qualitative study, we analyzed the effects of adoption of PRG-based payments for hospitals that replaced PD in Israel through the perspectives of hospital managers and physicians. We found many (unintended) consequences that go beyond the initial objectives of the reform (improve fairness of hospital payment; and strengthen the capacity of the MoH to supervise and control hospital activities). We found two main effects: organizational changes and responses to economic incentives. We also found important moderating factors that inhibited potential effects of the payment reform. Our study has important implications for researchers and policy-makers in many countries working on introducing or reforming DRG-based hospital payment systems.^26,27^

First, our results are in line with the existing theoretical literature on the effects of DRG-based hospital payments, which suggests that these payments create incentives to increase volumes of care, shorten ALoS, and select patients or procedures.^3,11,28^ Our findings illustrate from the perspective of hospital managers and physicians that these incentives exist (under certain conditions) and influence organizational and clinical discourse and decision-making, and that hospitals changed admission and treatment policies. Similarly, in line with the original intention of the development of DRGs and in line with the aims of other countries introducing DRG-based payment systems,^3,29^ our study suggests that the PRGs indeed contributed to increased transparency. The literature on the effects of adoption of DRGs also mentions upcoding as an unintended consequence.^10,11^ Some of our interviewees suggested that the PRG reform led to more precision in coding procedures and diagnosis, in order to increase revenues per case. Yet, we did not hear about upcoding, probably because the structure of PRG-based payments creates fewer opportunities for upcoding compared with DRGs. This is because PRGs depend only on procedures and not on diagnoses, which means that adding additional diagnoses does not increase the PRG tariff.

Second, our findings shed light on possible explanations for some of the inconclusive results of previous empirical studies on the effects of DRG-based payment systems on efficiency and quality of care.^10^ Recent studies have shown that hospitals in different countries react differently to the adoption of DRGs and subsequent changes in prices. For example, in Israel the introduction of PRGs had no significant impact on ALoS and volumes of care at the ward (aggregate) level,^9^ but in England volumes of care increased and ALoS decreased slightly.^30,31^ In Italy, volumes of care increased for surgical but not for medical patients^32^ while in Norway, volumes of care increased for medical but not for surgical patients.^33^ Our finding that profitability of individual PRGs determines the incentive to increase (or decrease) activity and that this incentive is moderated by other co-existing payment systems and other organizational constraints, may explain the (modest) effects in Israel – and potentially in other countries. This means that policy-makers have to carefully consider the context and the role of DRG-based payment within the overall hospital payment system, when introducing DRGs or reforming existing systems. Other potential explanations for different effects of DRG-based payment in different countries can be the (public) context and the role of DRG-based payment within the structure of hospitals as organizations; the dependency on healthcare resources; or the complex range of considerations that coexist among hospital managers and physicians. English hospital managers reported similar moderating factors that “muted” the responses to the early impact of adoption of the English variant of DRGs in 2005.^18^

Third, this study adds to the literature on the effects of DRGs and its country-specific variants, as it illustrates the wide range of effects of payment reform and how they influence hospital behavior beyond economic aspects. Increased transparency, and a common new language that enables enhanced supervision, show that PRG-based payments worked as intended at the organizational level: these are well-known important tools used by managers to change behavior in organizations.^34-36^ In the Israeli PRG case, these are tools to communicate the new economic incentives to surgical ward directors aiming to change their decision-making and behavior in line with the CEOs’ and hospital’s new objectives. Similarly, in Finland^37^ and Canada,^20^ the engagement of physicians in the implementation of DRGs depended much on transparency. We found that lack of transparency in communicating underlying (economic) rationales for changes in the organization of hospitals may create resistance to change and unintended behavior of physicians, such as adverse risk selection of patients. This implies that it is important to be transparent when communicating decisions in hospitals to those affected by them.

Fourth, our study indicates that the effects of payment reforms may be more limited than expected (by many policy-makers and economists) because economic considerations are moderated and sometimes outweighed by other important considerations.^18,38^ Interviewed physicians and managers always reported that they would not respond to economic incentives if their actions would reduce quality of care and patient safety. Also, commitment to patients and their families were often reported by physicians and managers to be important considerations that influence decision-making. Again, this is in line with existing literature, which shows that financial incentives and performance measurement as external motivators might be effective only for certain (limited) organizational changes,^39,40^ while the commitment to the patient’s health benefit usually outweighs self-interest and profit.^41-43^ Therefore, it seems that intrinsic motivation, which relies on trust, information and transparency, is at least as important as financial incentives to initiate change.^44^

Our study has several limitations. First, we did not interview all agents in hospitals such as the nursing staff, allied medical professionals, and laboratory technicians. These other agents may have different perspectives about the effects of the PRG payment reform over hospitals’ activities, culture and responses. However, these other professionals have less decision-making power regarding hospitals’ activities. Moreover, we tried to focus our analysis on the reform’s effects on patient’s admission and treatment policies, and did not attempt to capture broader effects on hospitals. Second, only one interviewed was female. Most of the surgeons and managers in Israeli hospitals are male, and this was reflected in our sample of interviewees. However, the underpresentation of females might present an imbalanced perspective on the effects of the PRG reform. Third, we did not interview ward directors in wards that did not participate in the “PRG reform,” such as medical wards, which might have been indirectly affected by the reform. However, our objective was to analyze the impacts of the payment reform, and therefore interviewing directors and physicians in wards not directly affected by the reform was beyond the scope of this study. Another type of limitation is the preconceptions that the Israeli authors had about the PRG reform. RW, DG, ED, and YP expected Israeli hospitals to respond to the PRG expansion similarly to how other countries responded to DRG-based payments, and the initial codes were built based on these expectations. During the analysis we learned that each country has its particularities, therefore we modified the codes and categories several times to reflect a more trustworthy reality for the Israeli case. In addition, working with non-Israeli coauthors (WQ and RB) gave a broader perspective about the effects of the reform and allowed less biased analysis of the data.

## Conclusion

This is the first qualitative study that analyzed the effects of the adoption of PRGs in Israel, and one of the first to assess the adoption of DRG-like payment reforms from hospital workers’ perspectives. Hospital managers and physicians explained processes that led to economic and organizational changes arising from the payment reform, which could not otherwise be captured by quantitative studies. Moreover, qualitative data allowed us to unpack moderating factors that led to unexpected consequences and inhibited other potential responses to this reform.

When shaping payment reforms, policy-makers must take into consideration that economic incentives are a powerful tool to change provider behavior, but not the only one. To increase the likelihood of achieving the intended effects of payment reform, it is important to consider the various economic incentives of – often multiple – coexisting PMs, and to align the incentives with the extent to which hospitals have the necessary autonomy and the required resources available to respond to these incentives. In addition, the specific configuration of the DRG-based payment part of the payment system, ie, the accuracy (or fairness) of payment and the scope of payment, influence the strength of the incentives.^45,46^ Therefore, researchers should refrain from simplistic assumptions when conducting quantitative cross-country comparisons of the effects of DRG-based payment. Managers should pay attention to organizational factors that enable change, such as transparency, information, a common language, clear communication and engagement of stakeholders. Finally, a reform is more likely to be implemented as intended if it enhances intrinsic motivation of providers, for example, aligning the economic incentives with other (non-financial) considerations.

## Acknowledgments

This study was funded by the Israel National Institute for Health Policy Research (grant no. 77/2016). RW thanks Minerva Stiftung for the Fellowship that supported her work.

## Ethical issues

The research, methods and interview protocol were approved by the ethics committee of Ben-Gurion University of the Negev (approval no. 1580-1).

## Competing interests

Authors declare that they have no competing interests.

## Authors’ contributions

Conceptualization, methodology and funding acquisition: RW; Formal analysis: RW, WQ, ED, YP, RB, DG; Investigation: RW, ED, DG; Supervision: WQ, RB, DG; Validation: WQ, YP, RB, DG; Writing: RW, WQ, DG.

## Authors’ affiliations

^1^The Smokler Center for Health Policy Research, Myers-JDC-Brookdale Institute, Jerusalem, Israel. ^2^Department of Health Systems Management, School of Public Health, Faculty of Health Sciences, Ben-Gurion University of the Negev, Beer-Sheva, Israel. ^3^Department of Health Care Management, Faculty of Economics & Management, Technical University Berlin, Berlin, Germany. ^4^European Observatory on Health Systems and Policies, Brussels, Belgium. ^5^Maccabi Healthcare Services, Tel Aviv-Yafo, Israel.


Supplementary file 1 contains Interview Protocol.

## Key Messages

**Implications for policy makers**

- Economic incentives can be powerful tools to change provider behavior, but they are not the only ones, and their strength depends on the context.
- To increase the likelihood of achieving the intended effects of payment reforms, it is important to consider the various economic incentives of – often multiple – coexisting payment methods (PMs), and to align the incentives with the extent to which hospitals have the necessary autonomy and the required resources available (personnel, bed and operating room (OR) capacity, post-acute care) to respond to these incentives.
- The accuracy of payment (ie, payments are similar to costs) and the scope of payment (ie, what is included in the payment), influence the strength of the incentives.
- Managers should pay attention to organizational factors that facilitate change, such as transparency of finances and activities, the availability of information, a common language shared between management and physicians, clear communication and engagement of stakeholders.
- A reform that intends to affect hospital workers and physicians is more likely to be implemented as intended if it enhances intrinsic motivation of providers, for example, aligning the economic incentives with other (non-financial) considerations.

**Implications for public**

We interviewed Israeli hospital managers and physicians. They reported that the change in the hospital payment unit from ‘hospitalization-day’ to the ‘procedure’ between 2010 and 2014 led to important effects. For example, it created a new common language for physicians and managers to discuss how to improve the management of patients with similar procedures. It also led to more transparency of activities and finances. At the same time hospitals increased activities (in certain areas), reduced costs per procedure and performed more (profitable) procedures. However, managers and physicians also explained that the effects of the reform were less strong than one might have expected because hospitals have many different financial and non-financial objectives. The public nature of hospitals and the (un)availability of resources such as personnel, or bed and operating room (OR) capacity reduced hospitals’ ability to increase activity, while conflicting incentives related to multiple different payment mechanisms or inaccurate pricing reduced the incentive to do so.

## References

[1] Srivastava D, Müller M, Hewlett E. Better Ways to Pay for Health Care. Paris: OECD; 2016. 10.1787/9789264258211-en

[2] Ellis RP, McGuire TG (1996). Hospital response to prospective payment: moral hazard, selection, and practice-style effects. J Health Econ.

[3] Geissler A, Quentin W, Scheller-Kreinsen D, Busse R (2011). Introduction to DRGs in Europe: common objectives across different hospital systems.

[4] Brammli-Greenberg S, Waitzberg R, Perman V, Gamzu R (2016). Why and how did Israel adopt activity-based hospital payment? The procedure-related group incremental reform. Health Policy.

[5] Rosen B, Waitzberg R, Merkur S (2015). Israel: health system review. Health Syst Transit.

[6] OECD. Hospital beds. OECD Data; 2017. https://data.oecd.org/healtheqt/hospital-beds.htm. Accessed March 9, 2017.

[7] OECD. Frequently asked question, OECD Health statistics, 2017. http://www.oecd.org/health/health-statistics.htm. Accessed March 9, 2017.

[8] Ministry of Health. Financial Report of the medical centers for years 2016–2017 [Hebrew]. 2019: Available at: https://www.health.gov.il/publicationsfiles/financial-analysis-2016-2017-accessible.pdf. Accessed March 9, 2017.

[9] Waitzberg R, Quentin W, Daniels E (2019). The 2010 expansion of activity-based hospital payment in Israel: an evaluation of effects at the ward level. BMC Health Serv Res.

[10] Palmer KS, Agoritsas T, Martin D (2014). Activity-based funding of hospitals and its impact on mortality, readmission, discharge destination, severity of illness, and volume of care: a systematic review and meta-analysis. PLoS One.

[11] Cots F, Chiarello P, Salvador X, Castells X, Quentin W. DRG-based hospital payment: Intended and unintended consequences. In: Busse R, ed. Diagnosis-Related Groups in Europe: Moving Towards Transparency, Efficiency, and Quality in Hospitals. Maidenhead: Open University Press McGraw-Hill Education; 2011.

[12] Moreno-Serra R, Wagstaff A (2010). System-wide impacts of hospital payment reforms: evidence from Central and Eastern Europe and Central Asia. J Health Econ.

[13] Cavalieri M, Gitto L, Guccio C (2013). Reimbursement systems and quality of hospital care: an empirical analysis for Italy. Health Policy.

[14] Or Z (2014). Implementation of DRG Payment in France: issues and recent developments. Health Policy.

[15] Shmueli A, Intrator O, Israeli A (2002). The effects of introducing prospective payments to general hospitals on length of stay, quality of care, and hospitals’ income: the early experience of Israel. Soc Sci Med.

[16] Baxter PE, Hewko SJ, Pfaff KA (2015). Leaders’ experiences and perceptions implementing activity-based funding and pay-for-performance hospital funding models: a systematic review. Health Policy.

[17] Finkler SA, Ward DM (2003). The case for the use of evidence-based management research for the control of hospital costs. Health Care Manage Rev.

[18] Sussex J, Farrar S (2009). Activity-based funding for National Health Service hospitals in England: managers’ experience and expectations. Eur J Health Econ.

[19] Palmer KS, Brown AD, Evans JM (2018). Standardising costs or standardising care? qualitative evaluation of the implementation and impact of a hospital funding reform in Ontario, Canada. Health Res Policy Syst.

[20] Baxter P, Cleghorn L, Alvarado K (2016). Quality-based procedures in Ontario: exploring health-care leaders’ responses. J Nurs Manag.

[21] Fässler M, Wild V, Clarinval C, Tschopp A, Faehnrich JA, Biller-Andorno N (2015). Impact of the DRG-based reimbursement system on patient care and professional practise: perspectives of Swiss hospital physicians. Swiss Med Wkly.

[22] Fourie C, Biller-Andorno N, Wild V (2014). Systematically evaluating the impact of diagnosis-related groups (DRGs) on health care delivery: a matrix of ethical implications. Health Policy.

[23] Wehkamp KH, Naegler H (2017). The commercialization of patient-related decision making in hospitals. Dtsch Arztebl Int.

[24] Braun V, Clarke V (2006). Using thematic analysis in psychology. Qual Res Psychol.

[25] Braun V, Clarke V (2014). What can “thematic analysis” offer health and wellbeing researchers?. Int J Qual Stud Health Well-being.

[26] Mathauer I, Wittenbecher F (2013). Hospital payment systems based on diagnosis-related groups: experiences in low- and middle-income countries. Bull World Health Organ.

[27] Busse R, Geissler A, Aaviksoo A (2013). Diagnosis related groups in Europe: moving towards transparency, efficiency, and quality in hospitals?. BMJ.

[28] Fetter RB (1991). Diagnosis related groups: understanding hospital performance. Interfaces.

[29] Fetter RB, Shin Y, Freeman JL, Averill RF, Thompson JD (1980). Case mix definition by diagnosis-related groups. Med Care.

[30] Parkinson B, Meacock R, Sutton M (2019). How do hospitals respond to price changes in emergency departments?. Health Econ.

[31] Farrar S, Yi D, Sutton M, Chalkley M, Sussex J, Scott A (2009). Has payment by results affected the way that English hospitals provide care? difference-in-differences analysis. BMJ.

[32] Verzulli R, Fiorentini G, Lippi Bruni M, Ugolini C (2017). Price changes in regulated healthcare markets: do public hospitals respond and how?. Health Econ.

[33] Januleviciute J, Askildsen JE, Kaarboe O, Siciliani L, Sutton M (2016). How do hospitals respond to price changes? evidence from Norway. Health Econ.

[34] Vainieri M, Ferrè F, Giacomelli G, Nuti S (2019). Explaining performance in health care: how and when top management competencies make the difference. Health Care Manage Rev.

[35] Kotter JP, Schlesinger LA (1989). Choosing strategies for change.

[36] Fiondella C, Macchioni R, Maffei M, Spanò R (2016). Successful changes in management accounting systems: a healthcare case study. Accounting Forum.

[37] Lehtonen T (2007). DRG-based prospective pricing and case-mix accounting—exploring the mechanisms of successful implementation. Manag Account Res.

[38] Wang V, Lee SY, Maciejewski ML (2015). Inertia in health care organizations: a case study of peritoneal dialysis services. Health Care Manage Rev.

[39] Pink DH. Drive: The Surprising Truth About What Motivates Us. Penguin Publishing Group; 2011.

[40] Flodgren G, Eccles MP, Shepperd S, Scott A, Parmelli E, Beyer FR (2011). An overview of reviews evaluating the effectiveness of financial incentives in changing healthcare professional behaviours and patient outcomes. Cochrane Database Syst Rev.

[41] Godager G, Wiesen D (2013). Profit or patients’ health benefit? exploring the heterogeneity in physician altruism. J Health Econ.

[42] Siciliani L (2009). Paying for performance and motivation crowding out. Econ Lett.

[43] Spoor C, Munro J (2003). Do budget-holding physicians respond to price? the case of fundholding in the UK. Health Serv Manage Res.

[44] Berenson RA, Rice T (2015). Beyond measurement and reward: methods of motivating quality improvement and accountability. Health Serv Res.

[45] Ellis RP, Miller MMK (2009). Provider payment methods and incentives.

[46] Quentin W, Scheller-Kreinsen D, Blümel M, Geissler A, Busse R (2013). Hospital payment based on diagnosis-related groups differs in Europe and holds lessons for the United States. Health Aff (Millwood).

