# Supplementary material for: Effects of Activity-Based Hospital Payments in Israel: A Qualitative Evaluation Focusing on the Perspectives of Hospital Managers and Physicians
Source: Int J Health Policy Manag. 2020 Apr 18;10(5):244–54. doi: 10.34172/ijhpm.2020.51 (PMC9056186; doi:10.34172/ijhpm.2020.51)
Supplement: Supplementary file 1 — contains Interview Protocol. [file ijhpm-10-244-s001.pdf]

Supplementary file 1. Interview Protocol

General introduction

• Thank you for agreeing to be interviewed and to contribute to our research.

• My name is ... and I work ...

• The subject of the study is payments to hospitals. Between 2010 and 2014, the Ministry of Health changed the method of payment - from payment by length of hospitalization to payment according to a procedure, which is a bulk that includes all the costs of the procedure. Regardless of length of hospitalization.

• The purpose of the study is to learn how this change affected hospitals' activity, and particularly how hospital workers live and feel about this change.

• During the interview, we will ask you to share your experiences and insights on the subject, and any matter that appears to you is relevant to the various issues.

• We received approval from the Ethics Committee to conduct the research. We are therefore obliged to request your approval and signature on the informed consent form.

• We would also appreciate your permission to record the interview because we will not be able to record everything.

• The length of the interview is about an hour.

• We guarantee complete confidentiality regarding your personal identity at all stages of the research.

• You are free to stop the interview at any stage you wish and you don’t have to answer to all the questions.

Questions for hospital Managers/CEOs (chief executive officers)

1. How old are you? How long have you been on this duty/position?
2. How did the change in payment method, from per diem to DRGs (diagnosis-related groups), affect your work as hospital manager? And how did it affect this hospital’s activities?
3. Following the change, what new messages did you convey to ward directors? And to the management of the operating rooms? To which wards did you convey new messages?
4. How do you convey your messages? With what techniques?
5. Is it difficult to convey the messages?

6. How did the relevant departments respond to the messages or instructions you gave?

7. How do you monitor the wards?

8. How did the change in payment method affect the negotiations between the hospital and the health plans (the agreements and discounts with the health plans)?

9. Has the change affected the relationship between you and the ward directors or the doctors?

10. Is there something that I didn't ask and I should have? Or something that I should be paying attention to? Or do you want to add anything?

Questions for ward directors

1. How old are you? How long have you been on this duty/position?

2. How did the change in payment method, from per diem to DRGs, affect your work as ward director? And how did it affect this ward's activities?

3. What new messages did you receive from the hospital manager?

4. What new messages do you convey to the doctors in this ward?

4. How do you convey your messages? With what techniques?

6. How did the doctors respond to the messages or instructions you gave?

Probe: Is it difficult to convey the messages?

7. How did the change affect the work here?

8. Did the change affect the relationship between yourself and the doctors?

9. Is there something that I didn't ask and I should have? Or something that I should be paying attention to? Or do you want to add anything?

Questions for doctors

1. How old are you? How long have you been on this duty/position?

2. Who determines which patient is operated, what procedures will be performed, and when patients are discharged?

3. Did what you just describe change since you started to work here?

4. Has there been a change in the prioritization of procedures in the ward? Has the work schedule changed?

(Probe: Are there any after-hours activities in the ward? If yes, was there any change in the type of procedures performed after-hours?)

5. What change occurred on your autonomy? For example, the type of considerations and decisions you take?

6. Does someone measure your performance? What is measured?

7. How does everything you have said so far, along with the change in the payment method, are expressed in management messages or expectations?

(Probe: for example, coding of procedures, clinical guidelines, use of certain devices or materials).

8. How does the hospital measure success? And how does the hospital or the ward manager give you feedback regarding your success?

9. (If there is time left: how has the relation between you and the management of the ward and the hospital change?)

10. Is there something that I didn't ask and I should have? Or something that I should be paying attention to? Or do you want to add anything?

Questions for Chief Financial Officers

1. How old are you? How long have you been on this duty/position?

2. How did the change in payment method, from per diem to DRGs, affect your work as CFO (chief financial officer)? And how did it affect this hospital's activities?

3. Following the change, what new messages did you convey to ward directors? Or to the CEO?

4. To which wards?

5. How do you convey your messages? With what techniques?

6. Is it difficult to convey the messages?

7. How did the relevant departments respond to the messages or instructions you gave?

8. How do you monitor the wards?

9. How did the change affect the negotiations between the funds (the agreements between the funds)?

10. How did the change in payment method affect the negotiations between the hospital and the health plans (the agreements and discounts with the health plans)?

11. Has the change affected the relationship between you and the ward directors or the CEO?

12. Is there something that I didn't ask and I should have? Or something that I should be paying attention to? Or do you want to add anything?
